# A Plant-Derived Remedy for Repair of Infarcted Heart

**DOI:** 10.1371/journal.pone.0004461

**Published:** 2009-02-14

**Authors:** Lei Cheng, Hao Chen, Xinsheng Yao, Guoqing Qi, Hongwei Liu, Kwongman Lee, Kaho Lee, Jieting Zhang, Shihui Chen, Xiaoli Lin, Wenchao Zhao, Jiankuan Li, Ming Li

**Affiliations:** 1 Key Laboratory for Regenerative Medicine of Ministry of Education of China, The Chinese University of Hong Kong, Hong Kong SAR, China; 2 Li Ka Shing Institute of Health Sciences, The Chinese University of Hong Kong, Hong Kong SAR, China; 3 Department of Anatomy, Faculty of Medicine, The Chinese University of Hong Kong, Hong Kong SAR, China; 4 Department of Cardiology, The First Affiliated Hospital, Hebei Medical University, Shijiazhuang, China; 5 Department of Pharmacology, Jian University, Guangzhou, China; 6 Key Laboratory of Systematic Mycology and Lichenology, Institute of Microbiology, Chinese Academy of Sciences, Chaoyang District, Beijing, China; University of Cincinnati, United States of America

## Abstract

**Background:**

Myocardial infarction (MI) due to coronary artery disease remains one of the leading causes of premature death. Replacement of infarcted heart tissue with regenerating myocardium from endogenous progenitor pools or exogenously introduced stem cells remains a therapeutic ideal. Their impracticality mainly lies in their low efficiency in cardiogenic differentiation (CD). Our recent studies with an acute MI animal model have already demonstrated the therapeutic effect of the MeOH extract of *Geum japonicum* (EGJ), providing clear evidence of myocardial regeneration.

**Methods and Findings:**

The present study further isolated the active component contained in EGJ using bioassay-guided isolation and investigated its efficacy in the treatment of infarcted heart in animal MI models. We demonstrated that substantial repair of infarcted heart in animal MI models by EGJ can be mimicked by the isolated candidate compound (cardiogenin) in MI animal models. Clear evidence of newly regenerated endogenous mesenchymal stem cells (MSCs) derived cardiomyocytes was observed throughout the infarct zone, accompanied by significantly improved functional performance of the heart. Transplantation of MSCs pretreated with EGJ or cardiogenin into a MI animal model also resulted in substantial regeneration of functional myocardium, implying that the activated MSCs carry all the necessary blueprints for myocardial regeneration. Signaling pathways specific to cell survival, CD identified in embryonic heart induction and angiogenesis were activated in both cardiogenin-treated MSCs and cardiogenin-induced regenerating myocardium.

**Conclusions:**

This study has demonstrated the therapeutic effects of cardiogenin in infarcted heart repair, and identified the associated signalling pathways for effective cardiogenic differentiation of MSCs, cell survival and angiogenesis. These findings should enable new treatment strategies for MI to be developed immediately.

## Introduction

Despite significant therapeutic advances, replacement of infarcted heart tissue with regenerating myocardium remains a therapeutic ideal as it is almost impossible for adult cardiomyocytes to repopulate [1]–[8]. The recent observation that a population of myocytes within the myocardium can replicate after infarction [9] has triggered an intense search for progenitor cells that can replace damaged myocardium. Transplantation of cardiomyocytes or myoblasts have both failed to reconstitute functional myocardium [3]–[5]. Transplantation of adult bone marrow (BM) derived MSCs following MI has been shown to produce some cardiomyogenesis, but majority of the newly regenerated cardiomyocytes appeared to be scattered along the less-ischemic border zone [7], [8]. This signifies that the transplanted MSCs had insufficient CD to form the integrated units necessary for regenerating functional myocardium. Considerable efforts have therefore been focused on searching for new ways to enhance cardiogenic differentiation efficiency (CDE) from a variety of potential progenitor cells, including embryonic stem cells, hemotopoietic stem cells and MSCs, since this is essential for myocardial regeneration as a viable and practical therapy for MI. However, the molecular mechanisms by which candidate progenitors can be stimulated to differentiate into cardiomyocytes remain largely unknown. An alternative solution is to search for molecules that can devise mechanisms which increase CDE in endogenous candidate progenitors.

In search of molecules promoting CDE, it is surprising to learn that the Miao, an ethnic group in China's Guizhou province, live significantly longer than other ethnic groups inhabiting the same region. A possible explanation for their longevity is that for centuries they have used their own kind of traditional medicine, which differs considerably from traditional Chinese medicine. We therefore screened the herbs frequently used in Miao medicine for treating common diseases that might possibly contribute to their longevity. One potential candidate was *Geum japonicum*, a herb used as a diuretic and an astringent [10] in Chinese medicine, but is also one of the commonly used herbs in many Miao medicine formulas. Our recent studies with an acute MI animal model have already demonstrated the therapeutic effect of EGJ, providing clear evidence of myocardial regeneration [11]. These findings have prompted the present investigation for the identification and isolation of the active components of EGJ, demonstration of its therapeutic effect for MI and elucidation of possible underlying signaling mechanisms.

## Methods

### Ethics Statement

All animal experiment protocols conformed to the Guide for the Care and Use of Laboratory Animals published by the U.S. National Institutes of Health, and approved by the Animal Experimental Ethical Committee of The Chinese University of Hong Kong.

### Bio-assay Guided Isolation of Cardiogenin from *Geum Japonicum*

**Figure 1:**
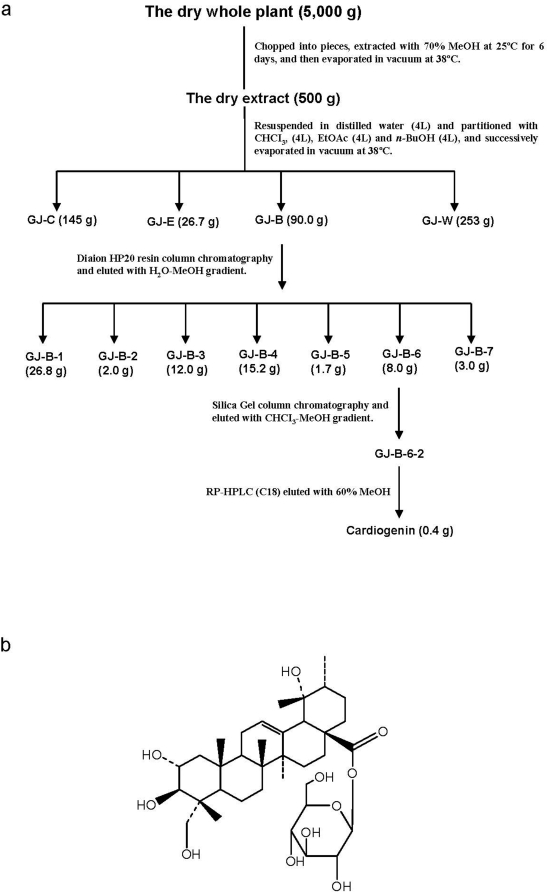
Bioassay-guided isolation of cardiogenin and its chemical structure. a, Diagram of bio-assay guided isolation of cardiogenin from *Geum japonicum*. ∼10% EGJ was extracted from the dried whole plant. The *n*-butanol fraction (GJ-B) from EGJ (18%) showed cardiogenic activity. Fraction 6 (GJ-B-6) was isolated from GJ-B by Diaion HP20 resin column chromatography and was shown to enhance myocardial regeneration. A cardiogenic fraction (GJ-B-6-2) was further isolated from GJ-B-6 using Silica gel column chromatography; and finally one cardiogenic compound (cardiogenin) was isolated from GJ-B-6-2 using RP-HPLC (C18). The recovery rate of the cardiogenin from GJ-B-6 or the dried whole plant is ∼0.5% or 0.8‰. b, Chemical structure of the isolated active component (chemically named Niga-ichigoside F1).

*Geum japonicum* collected from the Guizhou Province of China in August was dried, cut and percolated with 70% MeOH at room temperature for 6 days. The EGJ was produced as previously described [11]. The cardiogenic compounds was isolated from EGJ using a bio-assay guided strategy. As shown in the flow chart (Fig. 1), the isolation of the cardiogenic compound (cardiogenin) was attained by the following procedures: (i) the EGJ powder showing significant cardiogenic activity in MI animal model was prepared; (ii) the EGJ was suspended and partitioned with H_2_O and successively partitioned with CHCl_3_, EtOAc and *n*-BuOH respectively, whereby all 3 fractions were evaporated under reduced pressure (38°C) and yielded 3 different fractions; (iii) the cardiogenic-promoting effect of the three fractions was tested with MI animal models and it was found that the *n*-butanol fraction significantly enhanced myocardial regeneration in infarcted hearts; (iv) the *n*-butanol fraction was applied on a column of Diaion HP20 equilibrated with 10% MeOH and eluted with increasing concentration of MeOH in H_2_O, resolving 7 fractions; (v) activity testing in stimulating myocardial regeneration in a MI animal model demonstrated that fraction 6 was active in promoting myocardial regeneration *in vivo* while none of the other fractions together produced a significant cardiogenic effect, indicating that fraction 6 contained the active component responsible for the observed cardiogenic-enhancing effect of EGJ; (vi) further separation of fraction 6 by Silica Gel column chromatography isolated a major compound (C_36_H_58_O_11_) with proven *in vitro* and *in vivo* cardiogenic activity, herein termed cardiogenin. The chemical structure of cardiogenin was elucidated by NMR and MS analyses and compared with databases (ChemFinder, ChemACX Pro and ChemACX-SC Pro) and previous publications.

### MI Animal Model, Treatment Protocol and Assessment

MI was induced in male Sprague-Dawley rats (300∼350 g) by permanent ligation of the left anterior descending (LAD) coronary artery as previously described [11]. Each rat was anesthetized with intraperitoneal pentobarbital (50 mg/kg), intubated, and mechanically ventilated with room air using a Harvard ventilator (model 683). EGJ (300 mg/kg) or cardiogenin (0.3 mg/kg) dissolved in 5% DMSO (0.5 ml) was orally administrated to MI rats 1 day (acute) or 2 weeks (old) post ligation, daily for 2 weeks with equivalent amount of 5% DMSO to the sham-treated rats. For sham-operated rats (n = 8), thoracotomy was performed without LAD ligation.

Animals were sacrificed after final echocardiography measurements were recorded on day 7 and 14 post operations. The hearts of the sacrificed rats were removed and washed with PBS. All the specimens harvested were either sectioned for histological and immunohistochemical analyses or used for Western blot and DNA microarray studies. For histological examination, the left ventricles of the experimental rats sacrificed on day 7 and 14 were removed and cut from apex-to-base in 3 transverse slices and embedded in paraffin. The infarct size and vascular density in infarct zones were measured accordingly. Infarct size was expressed as a percentage of the total left ventricular area. To quantify the average number of capillaries in central infarct zones, 16 sections from within the central infarct zone of each heart were analysed, in which capillaries were counted and averaged in 6 randomly selected high power view fields (HPF, 40×) on each section. Blinded capillary counts were performed on 6–8 hearts per group by two investigators. The results were expressed as mean±SD capillaries per HPF. The detailed procedures for identification of the regenerating cardiomyocytes and myocardium; determination of the total volume of regenerating myocardium for hearts in each group; and related immunohistochemical analyses conducted in this study have been described in our previous report [11].

### Construction of Myosin Light Chain 2v-Promoted EGFP (MLC-GFP) Plasmid and its Transfection to MSCs

A recombinant expression vector was constructed by cloning a 2.7 kb HindIII–EcoRI fragment of the rat MLC-2v promoter region [5] into the HindIII–EcoRI site of pEGFP-1 plasmid (Clontech, Palo Alto, CA) to form a MLC-GFP plasmid, allowing GFP expression to be controlled by the MLC-2v promoter. To validate the construction, the pEGFP-1 plasmid and the constructed MLC-GFP plasmid with rat MLC-2v promoter were transfected to MSCs respectively. The non-transfected MSCs, transfected MSCs with a pEGFP-1 plasmid, and MSCs with a MLC-GFP plasmid were then treated with cardiogenin respectively. The results derived from this validation experiment demonstrated that (i) although cardiogenin-treated non-transfected MSCs showed an elongated phenotype compared to non-phenotypic changes in sham-treated cells, no GFP expression was observed, indicating that no endogenous GFP was stimulated; (ii) although cardiogenin-treated and sham-treated pEGFP-1 transfected MSCs underwent different phenotypic changes, both the cardiogenin-treated and sham-treated cells expressed GFP; (iii) a much higher percentage of cardiogenin-treated MLC-GFP plasmid transfected MSCs expressed GFP after 7 days treatment compared to a very low percentage of GFP expression observed in the sham-treated cells. The latter is probably due to unspecific leakage of the plasmid construction. This plasmid also contains the neomycin-resistance gene to enable selection of permanently transfected clones. The MLC-GFP plasmid was transfected into MSCs by liposomal transfection. After 24 h when cells are ∼90% confluent, a mixture containing 2.5 µg of plasmid DNA and 5 µl of Lipofectamine™ 2000 transfection reagent (Invitrogen) in OPTI-MEM^R^I (Invitrogen) were added to each 35 mm culture dish. After selection with 600 µg/ml of G418 for 4 weeks, stably transfected colonies were cloned and pooled. EGFP fluorescence was observed under a fluorescence microscope (Olympus TMD300, Tokyo, Japan).

### Assessment of EGJ/Cardiogenin-induced CDE of MSC

The tibias/femur bones of rats were removed and the BM was flushed out of the bones with alpha IMDM culture medium. The BM was mixed well and centrifuged at 1,500 rpm for 5 min. The cell pellet was suspended with 3 ml culture medium. The cell suspension was carefully put on 4 ml Ficoll solution to minimize disturbance and centrifuged at 200 rpm for 30 min. The second layer was transferred into a tube and washed twice with PBS to remove Ficoll (1,200 rpm for 5 min.). The cell pellet was resuspended in IMDM culture medium containing 10% heat inactivated FBS (GIBCO) and 1% penicillin/streptomycin antibiotic mixture and used for *in vitro* and *in vivo* studies. Non-adherent cells were discarded after 24 h culturing. The adherent cells were cultured by changing medium every 3 days and the cells became nearly confluent after 14 days culture. The MSCs were cultured with EGJ (60 µg/ml IMDM culture medium) or cardiogenin (10 µg/ml) for 3–4 and 6–7 days to sequentially activate cardiogenic specific genes' expression and morphology transition. The time-dependent expression of MEF2, GATA4,myosin heavy chain (MHC) and actin was assessed by immunecytochemistry at different time points. Briefly, cultured cells were fixed with 4% paraformaldehyde in PBS for 15 min and permeabilized with 0.5% Triton X-100 for 15 min. Dilution of antibodies was as follows: mouse monoclonal antibodies specific to MEF2 (1∶500), GATA4 (1∶300), MHC (1∶600) and actin (1∶500) (all from Santa Cruz). Secondary antibodies were goat anti-mouse IgG antibodies-conjugated with peroxides or Fluorophore (DyeMer™ 488/630 and 496/520) (Molecular Probe). The nuclei were stained with DAPI. For cell transplantation, both the cardiogenin-pretreated MSCs and the sham-treated MSCs were labeled with CM-DiI on day 3 of the cultures [4] prior to transplantation. CD associated morphological transition of MSCs was also examined by phase-contrast microscopic and immunocytochemical studies.

### Transplantation of Cardiogenin-pretreated MSCs and Assessment of Its Effect on Myocardial Regeneration

MI was induced by permanent ligation of LAD coronary artery of 24 rats as previously described [11]. For cell transplantation, the 5×10^6^ DiI-labeled cardiogenin-pretreated MSCs suspended (0.1 ml) in saline were injected into the blood stream of the 12 newly-induced MI rats in the test group. In parallel, the sham-treated group (n = 12) was injected with an equivalent amount of sham-pretreated DiI-labeled MSCs suspended in saline at the same injection site. Six experimental rats of both groups were sacrificed on day 7 and 14 respectively. The hearts of the sacrificed rats were removed, washed with PBS and photographed respectively. All specimens harvested were paraffin embedded and sectioned for tracing the signals of DiI florescence and examination of infarct size and myocardial regeneration. Colocalization of DiI label and cardiac-specific marker expression was examined and used to indicate CD of transplanted cells. The determinations of infarct size and capillary density in different groups of MI rats were carried out as described in our previous report [11].

The MSCs were cultured with cardiogenin (10 µg/ml) for 3 and 6 days to sequentially activate cardiogenic specific gene expressions. Prior to transplantation, both the cardiogenin-pretreated and the sham-pretreated control MSCs were labeled with CM-DiI on day 3 of the cultures. For cell transplantation, 5×10^6^ DiI-labeled cardiogenin- or sham-pretreated MSCs suspended (0.1 ml) in saline were injected into the blood stream through the tail vein immediately after LAD ligation in test and sham-treated groups respectively.

### Echocardiography Assessment of Heart Function

Echocardiography was recorded in all experimental subjects before and after EGJ/cardiogenin treatment, under controlled anesthesia, using a S10-MHz phased-array transducer and GE VingMed Vivid 7 system. M-mode tracing and 2D echocardiography images were recorded from the parasternal long- and short-axis views. Short-axis view was at the papillary muscles level. Left ventricular (LV) end-systolic and end-diastolic dimensions, as well as systolic and diastolic wall thickness were measured from the M-mode tracings by using the leading-edge convention of the American Society of Echocardiography. For each M-mode measurement, at least three consecutive cardiac cycles were sampled. LV end-diastolic (LVEDA) and end-systolic (LVESA) areas were planimetered from the parasternal long axis. LV end-diastolic and end-systolic volumes (LVEDV and LVESV) were calculated by the M-mode method. LV ejection fraction (LVEF) and fractional shortening (LVFS) were derived from LV cross-sectional area in 2D short-axis view: LVEF = [(LVEDV−LVESV)/LVEDV]×100% and LVFS = [(LVEDA−LVESA)/LVEDA]×100%. Standard formulae were used for echocardiographic calculations [2]. All data were analyzed offline with software installed in the ultrasound system. All measured and calculated indexes were presented as the average of three to five consecutive measurements.

### Signaling Pathway-specific Gene Expression Profiling and Genome Array Analyses

To determine the cellular origin of the regenerated myocardium and possible driving force involved in the processes of myocardial regeneration in infarcted hearts *in vivo* and CD of MSCs *in vitro*, signaling pathway-specific gene expression profiling analyses with Oligo GEarray® Rat Transduction Pathway Finder™ Microarray (SupperArray Bioscience Corporation) and GeneChip System Rat Genome 230 2.0 microarray (Affymetrix Inc. Santa Clara, CA) were performed with both cell and tissue samples. The cell samples were obtained from cultured MSCs treated with cardiogenin (5 µg/ml) and from MSCs treated with the equivalent amount of 5% DMSO at different durations of 12 h, 3, 7 and 14 days. The tissue samples were harvested from infarcted zones of EGJ-treated and sham-treated hearts, 3 days post treatment. All samples were sent to the Genome Center at the University of Hong Kong for analysis.

Total RNA was extracted from above prepared cell samples and infarcted heart tissue respectively. Each sample was homogenized using a Brinkmann PT 10/35 tissue homogenizer (Brinkmann Instruments, Inc, Westbury, NY) at medium speed for 30 to 60 sec. RNA was extracted using Trizol (Invitrogen) according to manufacturer's instructions. The total RNA concentration of each sample was determined by absorbance at 260 nm. All RNA preparations from both cell and tissue samples were treated with DNAase. The cRNA synthesis, labeling and amplification were performed according to the user manual of the TrueLabeling-AMP™ Linear RNA amplification kit. The synthesized cRNA were labeled with biotin (Roche). Prehybridization was performed in a hybridization oven at 60°C for 2 h; the biotin-labeled cRNA was then added into the prewarmed hybridization solution and hybridization continued at 60°C overnight. Chemiluminescent detection was then performed to capture the signal according to the manufacturer's instructions. Hybridization signals were detected by film exposure and analyzed by peptidylprolyl isomerase in the blots as an internal control for normalization.

### Inhibition of BMP-4 to Reduce CDE of MSC *in vitro* and *in vivo*

MSCs were cultured with cardiogenin (10 µg/ml) in the presence of a specific inhibitor of BMPs, noggin (10 ng/ml), for 4 days. The noggin-mediated suppression of cardiogenin-induced early- and further-CD was assessed by immunocytochemistry and Western blot analysis against MEF2 (on day 3 of culturing) and MHC (on day 6 of culturing), respectively. To confirm BMPs were involved in myocardial regeneration *in vivo*, on day 3 of the cultures, the cardiogenin-pretreated MSCs in the presence of noggin were labeled with CM-DiI in culture prior to transplantation. The labeled MSCs were injected into the blood stream of experimental rats with a newly made MI. Two and 3 weeks post infarction and MSC transplantation, whether the noggin mediated suppression of CD *in vitro* would affect myocardial regeneration *in vivo* was assessed.

### Statistics

All data were presented as mean±SD. Statistical significance for comparison between two measurements was determined by unpaired Two-tailed Student t-tests. Values of p<0.05 are considered to be significant.

## Results

### Isolation of Cardiogenin and Repair of Acute MI

**Figure 2:**
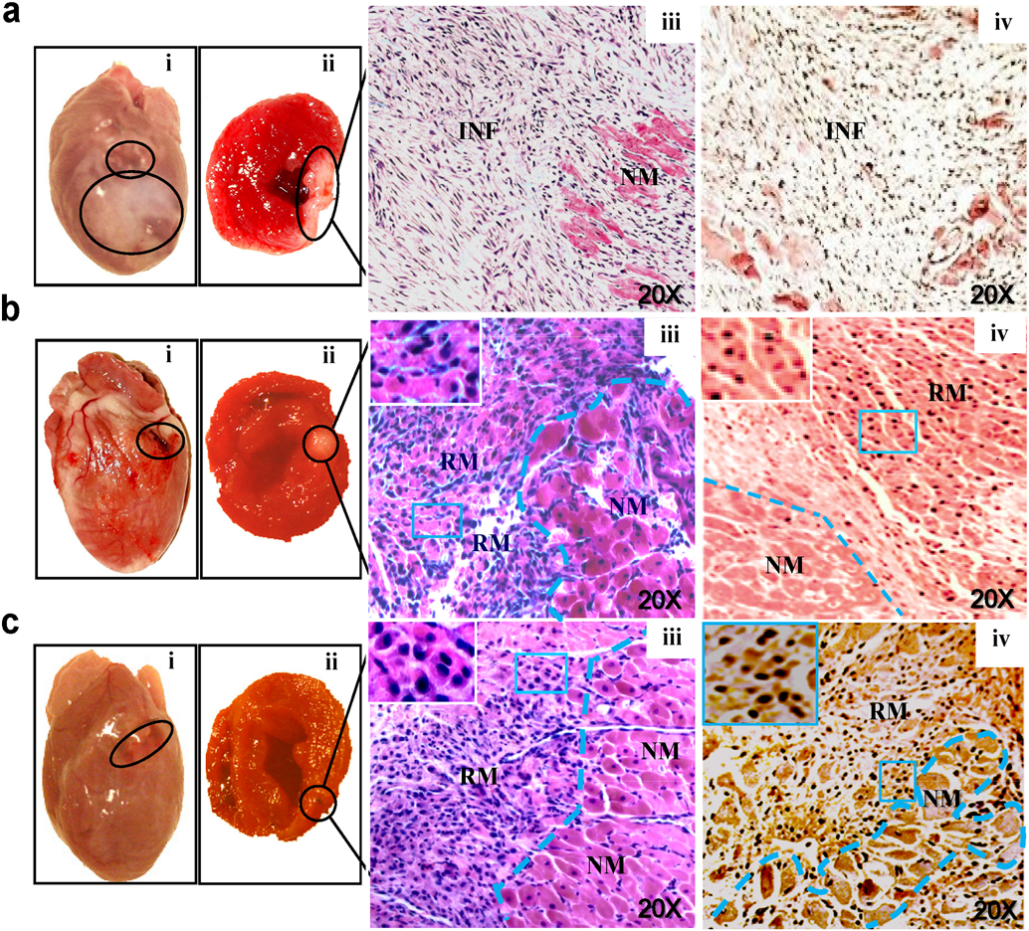
Significant repair of infarcted myocardia in acute MI rat model by EGJ/cardiogenin treatment. a, Sham-treated heart. Ligation site (upper circle) and a pale distal region (lower circle) (i). Heart cross-section showing the infarcted area (ii). HE stained section (iii). Ki67 (black nuclei) immuno-eosin-counter-stained section (iv), showing inflammatory cell infiltration and fibrous tissue replacement in the whole infarct zone (INF). EGJ− (b) and cardiogenin-treated (c) hearts with the ligation sites circled (b:i, c:i) and a corresponding heart cross-sections showing the infarcted area (b:ii, c:ii); HE stained sections (b:iii, c:iii). Ki67 immuno-eosin-counter-stained section (b:iv). Double Ki67 and MHC (brown) immuno-counter-stained sections (c:iv). Large number of regenerating myocytes (RM), distributed throughout the infarcted areas in treated hearts (b:iv, c:iv). However they are ∼1/3–1/4 times smaller than non-infarcted normal myocytes (NM). The regenerating cells formed bundles characteristic of typical myocardial morphology with Ki67 positively stained nuclei and MHC positively stained cytoplasm.

To identify and isolate the cardiogenic compound contained in EGJ, EGJ was attained by bio-assay guided fractionation (Fig. 1a). The chemical structure of the isolated cardiogenic-compound was determined by NMR and MS, and subsequently cross-referenced to databases (Fig. 1b). The therapeutic effect of the isolated compound, cardiogenin (chemically named Niga-ichigoside F1), was then compared with that of EGJ in the acute rat MI model to mimic acute MI in human patients. Although the area (4–6 mm in diameter) distal to the ligation site appeared pure white and thin due to ischemic necrosis in sham-treated hearts 14 days post infarction (Fig. 2a), the corresponding areas in both EGJ− (Fig. 2b) and cardiogenin-treated hearts (Fig. 2c) appeared red and the thickness of the ventricle and septal walls were well maintained. These observations are suggestive of possible infarction repair and myocardial regeneration. Histological examination revealed that the average infarct size, calculated as a percentage of the infarct volume of the total LV volume, in both EGJ and cardiogenin-treated hearts (n = 8 for each group, 18±6.9% and 20±7.3%, respectively) was ∼1/2–1/3 times smaller than that of sham-treated hearts (n = 8, 36±7.3%) (*p*<0.001) on day 14 post infarction. The fibrous replacement is characteristic throughout the infarct region of sham-treated hearts (Fig. 2a). In sharp contrast, the infarct zones in both EGJ− (Fig. 2b) and cardiogenin-treated hearts (Fig. 2c) were mostly covered by cells of typical cardiomyocyte-like morphology. However their sizes were ∼1/3–1/4 smaller than that of non-infarcted normal myocytes on the infarct border and distal regions, implying that these cardiomyocyte-like cells were newly regenerated. The cardiomyocyte-like cells clustered and organized into myocardial-like tissue throughout the infarct zone which were positively stained by antibodies specific to proliferation marker Ki67 and MHC (Fig. 2b:iv, c:iv) confirming that they are newly regenerated cardiomyocytes. These observations imply that both EGJ and cardiogenin have the capacity to enhance CDE, thereby accelerating MI repair.

**Figure 3:**
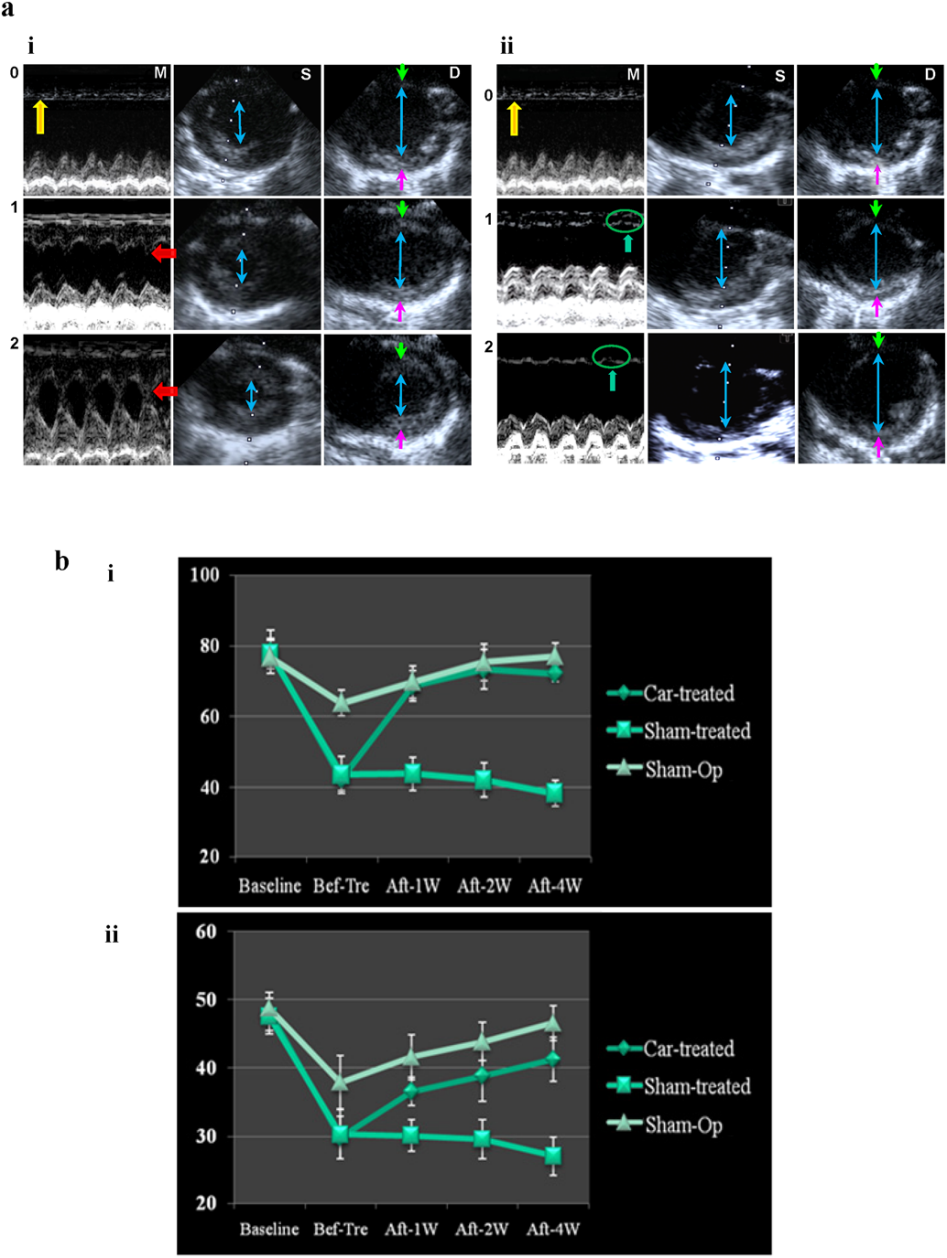
Echocardiography demonstration of improved heart function induced by cardiogenin treatment. a, Representative M-mode and end systolic/diastolic short-axis cross sectional images of the LV at the level of the papillary muscle of cardiogenin-treated (a:i) and of sham-treated (a:ii) MI hearts. M-mode measurements of a short-axis view showed motion of heart structure throughout several cycles (a:M); systolic short-axis view (a:S); and diastolic short-axis view (a:D). Images taken one day post ligation (a:0); 1 (a:1) and 2 weeks (a:2) treatment with cardiogenin or sham. One day ligation (a:0) resulted in anterior akinesis (yellow arrows) and marked LV dilation (blue arrowheads) in both groups of animals. One (a:i-1) and 2 (a:i-2) week cardiogenin treatment significantly and increasingly improved the LVAW motion (red arrow) and reduced systolic/diastolic (blue arrowheads) volumes. Sham-treated hearts showed progressive increases in systolic/diastolic volumes (a:ii-S,D, blue arrowheads). Systolic performance progressively worsened with anterior akinesis developing into paradoxical motion (a:ii-M, green arrows and circles). The LVAW thickness in cardiogenin-treated heart (a:i-S,D), (a:i-D, the space between the green and blue arrowheads) gradually increased, with non-visible changes of LV posterior wall thickness (the space between blue and pink arrowheads). Both the anterior and posterior LV wall thickness in sham-treated heart (a:ii-S,D) became increasingly thin. b, Echocardiography with LVEF (b:i) and LVFS (b:ii) demonstrating improved heart function with time by cardiogenin treatment (Car-treated). Sham-treated (Sham-treated) vs cardiogenin-treated hearts before ligation (baseline), 0–4 weeks after cardiogenin/sham treatment. Both graphs show regain of cardiac function in cardiogenin-treated but not sham-treated hearts (n = 8 for each group), in which p = 0.005 for week 1, p = 0.001 for weeks 2 and 4. Sham-Op refers to open-chest operation without LAD ligation.

To demonstrate that the significant cardiac regeneration in the cardiogenin-treated MI hearts was accompanied by progressively improved functional performance, echocardiography was performed at different time points before and after cardiogenin or sham (n = 8 for each group) treatment. It was found that the wall motion of the LV anterior wall (LVAW) in cardiogenin-treated hearts was gradually restored, while the LVAW in sham-treated hearts were thinned and akinetic, which developed into paradoxical motion 1–4 weeks post infarction (Fig. 3). Progressive restoration of the systolic/diastolic volume in cardiogenin-treated hearts was also evident (Fig. 3a). The time-dependent functional improvement in terms of LVEF and LVFS in the cardiogenin-treated group was significant (Fig. 3b).

### Cardiogenin Induced Substantial Repair of Old Infarcted Heart

To further demonstrate the therapeutic effect of cardiogenin on old infarcted hearts to mimic the commonest situation in patients, MI animals (n = 10) received oral administration of cardiogenin dissolved in 5%DMSO (0.3 mg/kg) or equivalent amount of 5% DMSO 2 weeks post LAD ligation for 2 weeks. Measurements taken before treatment and at 1, 2 and 4 weeks after cardiogenin or sham treatment in old MI rats (Table 1) showed that heart function was significantly improved with progressively restored LVAW motion (originally motionless at 2 weeks post ligation) and systolic/diastolic volume, similar to cardiogenin-treated acute MI hearts. Consistent with sham treatment in the acute MI model, LV systolic/diastolic volumes progressively increased in sham-treated old MI rats. Furthermore, at 4 weeks post infarction, systolic performance worsened with anterior wall akinesis which developed into paradoxical motion (Table 1). The time-dependent functional improvement in acute MI cardiogenin-treated hearts was also apparent in the old MI cardiogenin-treated hearts. The results obtained from acute and old MI experiments suggest that cardiogenin is an active component from EGJ responsible for the observed myocardial regeneration.

**Table 1:** Echocardiography evaluation of LV function in old MI rats.

Evaluations	Two weeks post MI prior to treatment		Post treatment		Post/prior%	
	Car	Sham	Car	Sham	Car	Sham
**LVEDD (nm)**			7.3±2.9*	8.0±2.1*	−8.7*	+1.3*
	8.0±2.2	7.9±2.3	6.8±2.6**	8.7±2.6**	−15.0**	+10.1**
			6.4±2.5***	9.1±2.8***	−20.0***	+15.2***
**LVESD (nm)**			4.8±2.5*	5.6±2.5*	−17.0*	−1.8*
	5.8±2.1	5.7±2.0	4.3±2.1**	6.3±2.3**	−25.8**	+10.5**
			3.8±2.2***	7.2±3.0***	−34.4***	+26.3***
			48.8±6.1*	41.8±5.3*	+21.7*	−1.9*
**LVEF**	40.1±4.8	42.6±5.1	60.1±6.6**	40.1±4.9**	+49.8**	−5.9**
			65.4±5.5***	37.3±5.0***	+63.0***	−12.4***
			32.2±4.3*	27.8±4.1*	+15.4*	−5.1*
**LVFS**	27.9±5.2	29.3±4.8	35.8±4.8**	27.6±4.8**	+28.3**	−5.8**
			39.1±5.5***	25.6±3.9***	+40.1***	−12.6***

LVESD: left ventricular end systolic diameter; LVEDD: left ventricular end diastolic diameter; LVEF: ejection fraction; LVFS: fraction shortening, *1; **2; ***4 weeks post treatment.

### Endogenous MSCs are Involved in Acute Myocardial Regeneration

**Figure 4:**
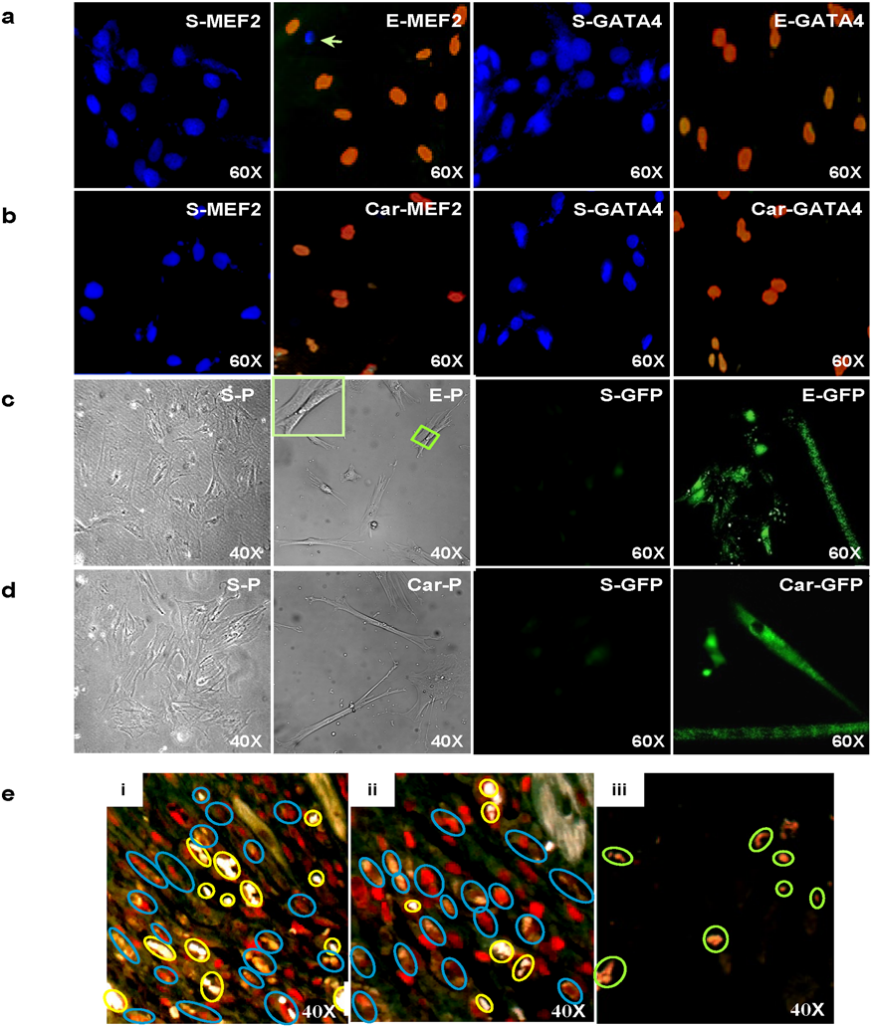
EGJ/cardiogenin-enhanced CDE of MSCs *in vitro* and *in vivo*. a, EGJ-treated and b, cardiogenin-treated MSCs double-stained with DAPI (blue nuclei) and specific early markers of CD, MEF2 (E-MEF2, Car-MEF2) and GATA4 (E-GATA4, Car-GATA4); with corresponding sham-treated MSCs stained positive for DAPI but negative for CD markers (S-MEF2, S-GATA4). Note the presence of a DAPI-positive but MEF2-negative nucleus among other MEF2-positive nuclei in EGJ-treated MSCs (E-MEF2, white arrow) is possibly due to a proportion of MSCs not committed to cardiac differentiation, which may also serve as a negative control. Three days post treatment, cells were immunocytochemically stained against MEF2 and GATA4 individually, and then the nuclei were stained blue with DAPI. Low magnification phase-contrast images and green fluorescent micrographs of EGJ-treated (c) and cardiogenin-treated (d) MSCs. Six days post treatment, both EGJ- and cardiogenin-treated MSCs were elongated with characteristic striated appearance (c:E-P, d:Car-P) and GFP-positive (c:E-GFP, d:Car-GFP) in contrast to the irregularly shaped MSCs and absence of green fluorescence in sham-treated cells (c:S-P, d:S-P, c:S-GFP, d:S-GFP). e, MEF2-immunostained and DiI-labeled heart tissues demonstrating *in vivo* CD of cadiogenin-activated MSCs at 7 days (i) and 14 days (ii) after transplantation to MI hearts. Sham-treated MSCs 14 days after transplantation (iii). Numerous DiI (orange) and MEF2 (red) colocalized cells bearing cardiomyocyte phenotype (blue circles) aligning in similar orientation with pre-existing cardiomyocytes were observed with many capillaries (i–ii, yellow circles) found throughout the infarct zone of cardiogenin-activated MSC-transplanted MI hearts. In sham-treated MSC transplanted MI hearts, only DiI-positive cells without MEF2 colocalization (iii, green circles) were observed scattering along the infarct zone.

To determine the particular cell type contributing to EGJ/cardiogenin-induced myocardial regeneration, genome array analysis was conducted to examine the expression of cell markers. Results showed MSC-specific markers including CD29, BST-1, SDF-1 and SDF-4, were detected in EGJ-treated tissues 3 days post infarction, indicating their MSC origin. We thus hypothesized that EGJ/cardiogenin could induce CD in MSCs at an efficient rate sufficient for myocardial regeneration. Hence the effect of EGJ/cardiogenin in inducing CD of MSCs *in vitro* was studied. MSCs cultured in medium containing EGJ (60 µg/ml) or cardiogenin (10 µg/ml) were stained positive for antibodies specific to two of the earliest cardiogenic lineage markers, MEF2 (>90%) and GATA4 (>70%), respectively (Fig. 4a and b), signifying their early commitment to CD. Phase-contrast microscopy demonstrated that treated MSCs differentiated into elongated cells, some of which showed a recognizable striated appearance, differing from the irregularly shaped undifferentiated MSCs in the sham-treated cells (Fig. 4c and d). To further confirm CD of MSCs, MLC-GFP-transfected MSCs were treated with EGJ (60 µg/ml) or cardiogenin (10 µg/ml) for 3 and 6 days. It was found that >70% and 50% of the MSCs became rod-shaped with green fluorescence, respectively (Fig. 4c and d), which can only be observed with the expression of cardiac-specific MLC. This phenotype verifies the differentiation of MSCs into cardiac progenitors. In sharp contrast, <6% of the sham-treated cells displayed green fluorescence on day 6. These results suggest that both EGJ and cardiogenin can significantly increase CDE in MSCs *in vitro*, which we hypothesized could be sufficient for myocardial regeneration *in vivo*.

### Cardiogenin-activated MSCs Repaired MI

To test whether increased CDE in progenitor cells alone could lead to significant myocardial regeneration for repair of infarcted hearts *in vivo*, DiI-labeled MSCs were pretreated with cardiogenin for 3 days and washed thoroughly to remove residual cardiogenin prior to injection of the cells into the blood stream of newly-induced MI rats. One week post infarction and cell transplantation, sectioned heart tissues were immunostained for MEF2, and results showed numerous DiI and MEF2 colocalized cells in MI hearts transplanted with cardiogenin-activated MSCs, confirming their donor cell origin and *in vivo* cardiomyogenic differentiation (Fig. 4e). Furthermore, these cells aligned in similar orientation with viable non-infarcted cardiomyocytes and distributed throughout the infarct zones. Additionally, significantly more MEF2-positive cells were detected at 14 days than at 7 days post-treatment (Fig. 4e). By contrast, much less DiI and MEF2 colocalized cells were observed in the hearts transplanted with sham-pretreated MSCs (Fig. 4e), possibly due to the low CDE in the sham-treated MSCs. Capillary density in the infarct area of hearts transplanted with cardiogenin-activated MSCs 7 days post transplantation was on average 13±3 per HPF (Fig. 4e), whereas only 5±2 vessels per HPF were in the infarct zone of hearts transplanted with sham-pretreated MSCs. The highly organized regenerating myocardium-like tissues in the hearts transplanted with cardiogenin-activated MSCs occupied on average 63±8.0% of the total infarct volume 7 days post treatment as compared to <8±3.6% in hearts receiving sham-treated MSCs. Collectively, these results suggest that the ability of EGJ/cardiogenin to repair infarcted heart could be due to their ability to increase CDE of endogenous MSCs.

### Signaling Pathways are Possibly Involved in Cardiogenin-mediated Myocardial Regeneration

**Figure 5:**
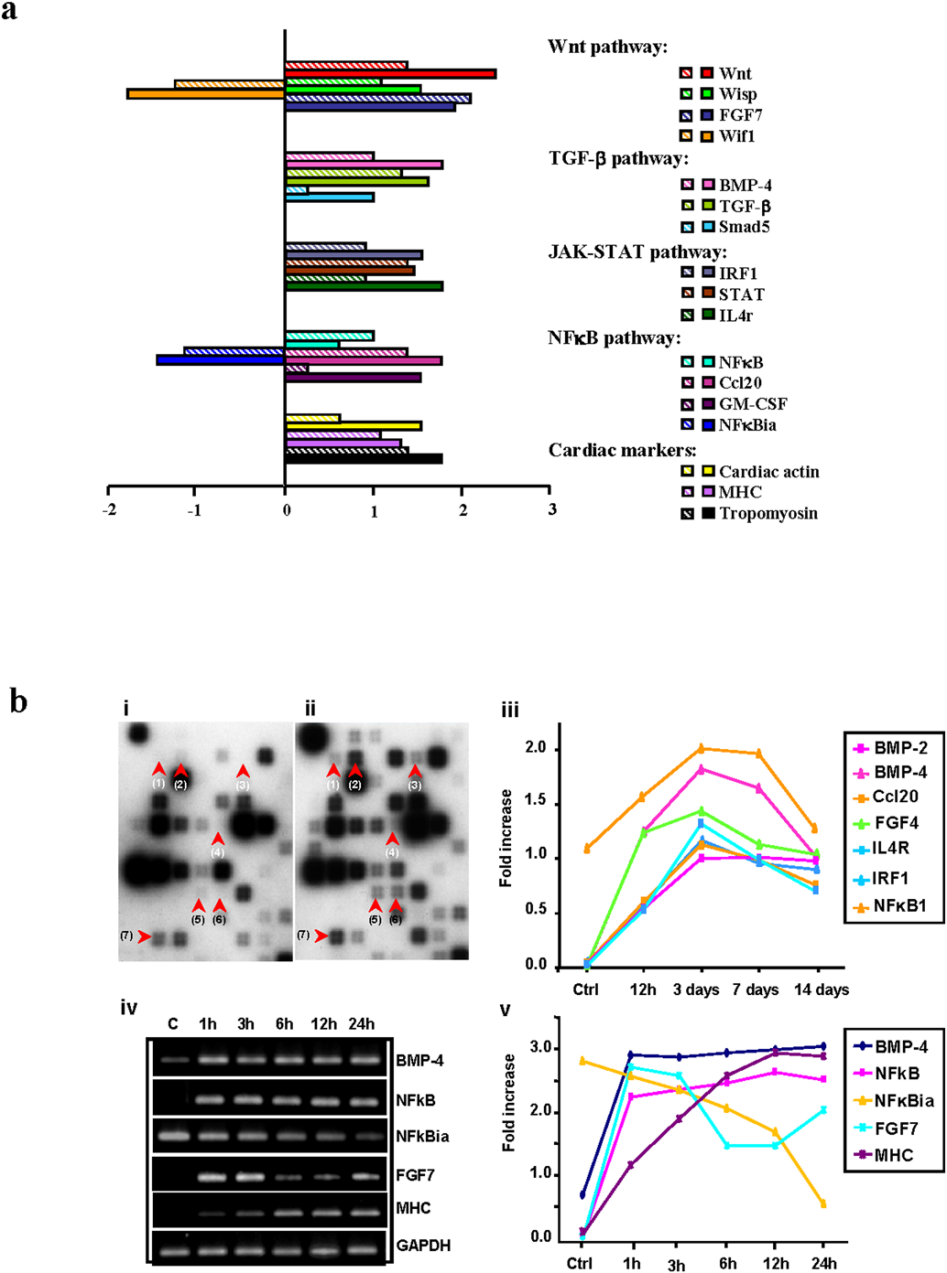
Activation of selective signaling pathways in EGJ-treated MI hearts or cardiogenin-treated MSCs. a, Signaling pathways including Wnt, TGF-β, JAK-STAT, and NFκB were found similarly activated/up-regulated in both cardiogenin-treated MSCs (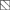) and EGJ-treated MI hearts (▪) on day 3 after treatment, indicating similar mechanism involved in both cardiogenin-enhanced CDE in MSCs and EGJ-induced myocardial regeneration. Some CD and cardiac tissue-specific markers were also detected. b, Representative pathway-specific DNA arrays obtained from MSCs before (i) and 3 days (ii) after cardiogenin treatment (10 µg/ml) with red arrowheads highlighting activation/upregulation of Wnt (4, FGF4), TGF-β (1, BMP-2; 2, BMP-4), JAK-STAT (5, IL4R; 6, IRF1) and NFκB (3, Ccl20; 7, NFκB1) pathways. The pathway-specific genes were expressed in a time-dependent manner, monitored over 12 h, 3, 7 and 14 days after cardiogenin treatment (iii). Representative RT-PCR (iv,v) confirmed time-dependent changes in expression levels of pathway-specific and CD-specific genes in MSCs induced by cardiogenin.

Activation of signaling pathways using Oligo GEarray® Rat Transduction Pathway Finder™ Microarray was used to determine the possible signaling pathways involved in mediating the therapeutic effect of EGJ/cardiogenin on CD and MI repair. The results showed that similar signaling pathways were activated in both cardiogenin-treated MSCs *in vitro* and EGJ-treated hearts *in vivo* 3 days post treatment (Fig. 5a). We then further examined time-dependent activation of these signaling pathways during cardiogenin-induced CD in MSCs using the same microarray. It was demonstrated that cardiogenin treatment of MSCs *in vitro* induced activation of several signaling pathways, including Wnt, TGF-β, JAK-STAT and NFκB pathways (Fig. 5b:i–v). Most of these signaling pathways were activated/upregulated 1 h after treatment, and kept increasing at 12 h, peaked on day 3, and declined on day 7 and 14 (Fig. 5b:iii). The time-dependent expression profiles of these pathway-specific key genes were further confirmed by RT-PCR analysis (Fig. 5b:iv and v). The activation of these four signaling pathways, which have been studied extensively and implicated in early heart induction during embryonic development [13], is consistent with their role in promoting CD in MSCs and myocardial regeneration in infarcted hearts treated with EGJ/cardiogenin.

**Figure 6:**
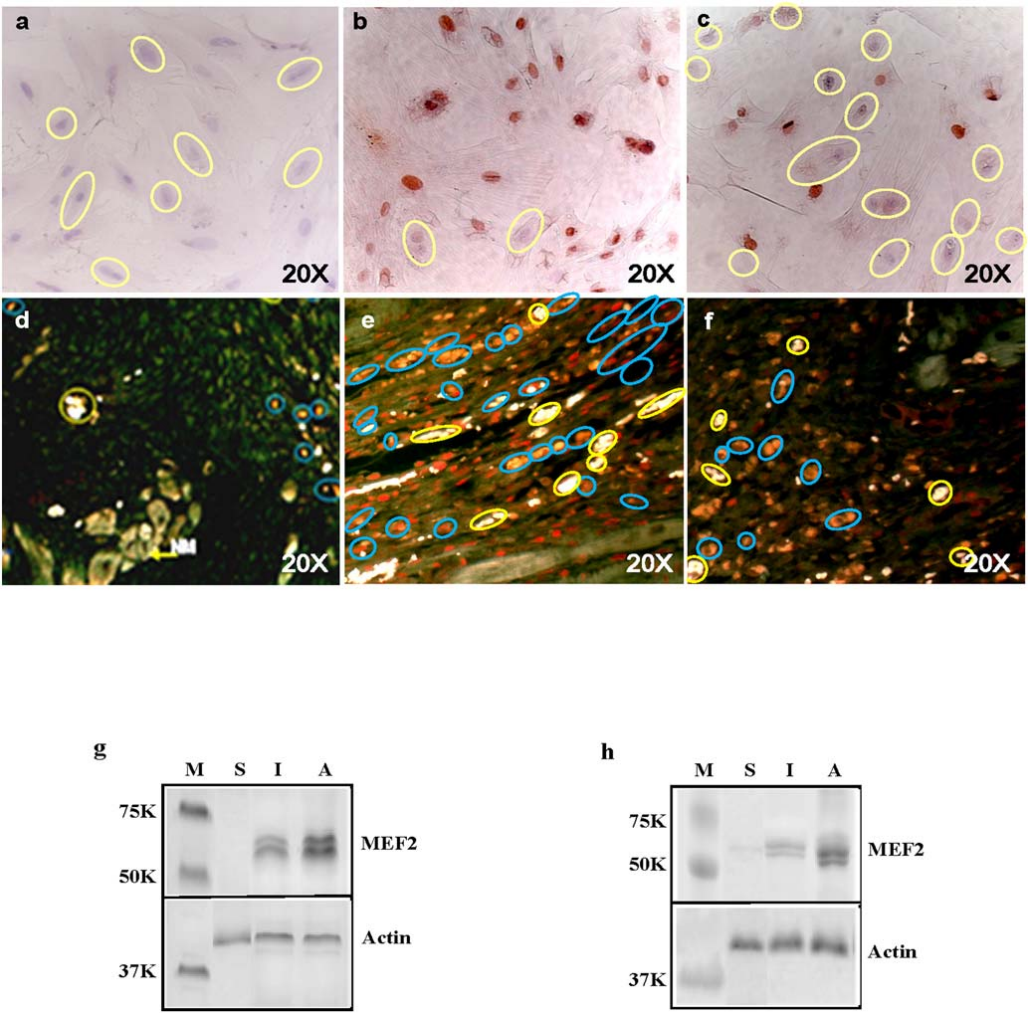
Suppressed CDE in MSCs by BMP-4 inhibition *in vitro* reduced cardiomyogenesis *in vivo*. Representative MEF2 immunostaining micrographs of sham-treated (a), cardiogenin-treated (b) and cardiogenin-noggin-treated (c) MSCs, showed different levels of MEF2 expression. There were only MEF2-negative cells in the sham-treated (circled in yellow). In sharp contrast, >90% of MEF2-positive cells (brown nuclei) with a few coexisting negatively stained nuclei were observed after 3 days culture with cardiogenin (10 µg/ml), and ∼50% of the MSCs treated with cardiogenin and noggin (10 ng/ml) were MEF2-negative (c). Representative MEF2-immunostained and DiI-labeled heart tissues demonstrating *in vivo* CD of cadiogenin-activated MSCs 7 days after transplantation were also observed. Note that many DiI (orange) and MEF2 (red) colocalized cells (circled in blue) and many coronary capillaries (circled in yellow) appeared throughout the whole infarct zone (e). Only a few DiI- and MEF2-positive cells were observed in the infarcted region of the MI hearts transplanted with sham-pretreated MSCs (d). In regard to cardiogenin-noggin-transplanted MSCs, although many DiI-positive cells were observed in the infarct zone, significantly less MEF2-positive cells were present. Western blots (g, h) were also performed from cell (a–c for g) and tissue (d–f for h) samples to verify the results in a–f. Bands were produced from M, molecular weight marker; S, sham-treated; I, noggin-inhibited; and A, cardiogenin-activated MSCs. MEF2 expression in the presence of noggin was ∼2.3 folds less than that in cardiogenin-activated MSCs *in vitro*. This quantification matches the observations in a–c. Western blot analysis (h) also matched immunohistochemistry results *in vivo* in which MEF2 expression in the presence of noggin (f) was ∼2.6 folds less than that in the absence of noggin (e).

BMP-4 of the TGF-β pathway was found in the present study to be one of the earliest genes maximally upregulated in both EGJ-induced myocardial regeneration and cardiogenin-induced CD in MSCs. In a previous publication, BMPs induced cardiac differentiation of P19CL6 *in vitro*, in which the cardiac differentiation was blocked by noggin, a specific inhibitor of BMPs [12]. Therefore, to verify the involvement of BMP-4 in CD of MSCs and MI repair, noggin (R and D Systems) was used to suppress cardiogenin-induced CD of MSCs. As shown in Figure 6, >90% of MSCs treated with cardiogenin for 3–4 days were found to be immunoreactive with MEF2 antibody (Fig. 6b), while <50% of the cardiogenin-treated MSCs in the presence of noggin (10 ng/ml) were MEF2-positive (Fig. 6c). These results indicated significant reduction in cardiogenin-enhanced CDE of MSCs, which was further confirmed by Western blot analysis. Where, the expression of MEF2 in cardiogenin-treated MSCs with noggin was indeed 2.3 folds less than that in the absence of noggin (Fig. 6g), confirming the inhibition of BMP signaling by noggin. Similarly, only ∼55% of the cardiogenin-activated MSCs in the presence of noggin was positively stained with antibody specific to MHC, while ∼95% of the MHC-positive cells was observed in the cardiogenin-treated MSCs without noggin. These results demonstrated that inhibition of BMP-4 by noggin leads to reduced cardiogenin-enhanced CDE of MSCs *in vitro*.

To test whether cardiogenin-induced cardiomyogenic differentiation *in vivo* could be suppressed by inhibition of BMP signaling, we further transplanted the cardiogenin-noggin-treated MSCs in a MI animal model. Approximately 80% of the donor MSCs were positively stained by MEF2-specific antibody in cardiogenin-activated MSCs that had been transplanted (Fig. 6e). By contrast, <30% of the donor MSCs treated with noggin were MEF2- positive (Fig. 6f), signifying that BMP suppression could reduce cardiomyogenic differentiation of the MSCs *in vivo*. Western blot analysis also showed results consistent with immunohistochemical results in that MEF2 expression in MI hearts transplanted with cardiogenin-activated MSCs was ∼2.6 folds more than that in those transplanted with cardiogenin-noggin-treated MSCs (Fig. 6h). Collectively, these results corroborate that inhibition of BMP-4 leads to reduced CDE of MSCs both *in vitro* and *in vivo*, which is perhaps due to the fact that activation of BMP signaling is one of the earliest events in CD, as demonstrated by the activation of signaling pathways, on which full activation of other pathways and CD may depend.

## Discussion

As a whole, this study has demonstrated that the therapeutic effect to repair infarcted hearts by EGJ or cardiogenin lies critically in their ability to enhance CDE of endogenous BM-derived MSCs, which otherwise would occur at a rate too low to be sufficient for myocardial regeneration. The regenerating cardiomyocytes observed in this study mainly originated from BM- derived MSCs. BM-derived haematopoietic stem cells may be excluded from being a possible contributor in myocardial regeneration, since a previous study of ours showed that haematopoietic stem cells could not be induced to undergo CD by EGJ/cardiogenin (unpublished data). The inability of haematopoietic stem cells to differentiate into myocardiocytes is consistent with other studies [14], [15].

It should be noted that recent studies have reported cardiomyocytes derived from transplanted human embryonic stem cells (hES). Although the findings are of great interest [16], [17], the use of hES may be limited by the potential formation of teratomas containing derivatives of all three germ layers and immune rejection [18]–[21]. Our present results have provided an alternative way to promote significant myocardial regeneration by a plant extract or its active compound. The activation of endogenous MSCs for repair of infarcted heart will eliminate possible teratoma formation and immune rejection that may be associated with the use of hES. More importantly, the application of EGJ/cardiogenin is convenient and safe compared to other treatment strategies which usually involve cell transplantation or gene manipulation that may potentially bring about many severe complications. Therefore, EGJ/cardiogenin should be considered for immediate development as a new treatment remedy for MI.

The present findings suggest that EGJ/cardiogenin essentially activates four major signaling pathways to a level sufficient for myocardial regeneration and repair of infarcted hearts. These signaling pathways appear to act coherently in a time-dependent manner to bring about CD, since inhibition of one of the earliest upregulated molecules, BMP-4, is able to reduce MI repair *in vivo* or CD of MSCs *in vitro*. Interestingly, BMPs has also been shown to stimulate CD from hES [22]. Together with the present findings, it implies that BMP-4 may be essential in CD regardless the cell types, hES or MSCs, from which cardiomyocytes are derived.

Evidence from this study also suggests possible involvement of other mechanisms elicited by EGJ/cardiogenin in repair of infarcted heart. The observed activation/upregulation of Akt1 [23], Bcl2 and cell survival associated pathways, such as JAK-STAT [24] and NFκB [25]–[28] pathways in both cardiogenin-activated MSCs and EGJ-treated MI hearts propose that EGJ/cardiogenin does have the ability to increase the survival potential of myocytes at risk along infarct rim and MSCs that migrate to the infarct zone. The observed numerous DiI-positive regenerating cardiomyocytes throughout the whole infarct zone of MI hearts transplanted with EGJ/cardiogenin-activated MSCs, as compared with a few scattered DiI-positive cells in sham MI hearts, could also be due to, at least partly, the increased survival potential of donor cells in the infarct zone.

In addition, many new vessels were observed in the whole infarct zone within 24 h in the treated, especially EGJ-treated, but not sham-treated MI hearts. Angiogenesis is required for the survival, trafficking and growth of cells necessary for the repair of infarcted heart, indicative of possible angiogenic action of cardiogenin as well as other possible angiogenic compound(s) contained in EGJ. The pathways of JAK-STAT and NFκB have been well documented to induce various angiogenic factors besides antiapoptotic proteins [24]–[26]. Thus, activation of these pathways in cardiogenin-pretreated MSCs may also promote angiogenesis, and therefore, promote the reconstitution of damaged coronary vessels and further prevent cell death of transplanted MSCs for efficient MI repair.

The demonstrated therapeutic effect of EGJ and cardiogenin in our animal MI model and enhanced CDE of MSCs *in vitro*, as well as the similar signaling pathways activated by EGJ and cardiogenin prove that cardiogenin is a major active compound responsible for significant repair of infarcted hearts. The identification of the chemical structure and therapeutic effect of cardiogenin, as well as an underlying mechanism involved, provide ground for immediate development of new treatment strategies for MI.
